# Tissue-Resident Mesenchymal Stem/stromal Cells (MSC) Modulate the Angiogenic Processes in Brain Arteriovenous Malformations (bAVM)

**DOI:** 10.1007/s12015-025-10937-1

**Published:** 2025-07-17

**Authors:** Claudia Alexandra Dumitru, Belal Neyazi, Tamer Ayberk Kaya, Klaus-Peter Stein, Ali Rashidi, Christian Mawrin, Ibrahim Erol Sandalcioglu

**Affiliations:** 1 Department of Neurosurgery, Otto-von-Guericke University, Magdeburg, Germany; 2 Department of Neuropathology, Otto-von-Guericke University, Magdeburg, Germany; 3 Leipziger Str. 44, 39120 Magdeburg, Germany

**Keywords:** Mesenchymal stem/stromal cells, Brain arteriovenous malformations, Angiogenesis, Endothelial-to-mesenchymal transition

## Abstract

**Background:**

Mesenchymal stem/stromal cells (MSCs) have been mainly studied in the context of cell-based therapy for a variety of medical conditions, including cerebrovascular diseases. However, the role of tissue-resident MSCs in the pathophysiology of cerebrovascular diseases in general and of brain arteriovenous malformation (bAVM) in particular is currently unknown, and was investigated in this study.

**Methods:**

Human bAVM tissues were used to identify MSCs in situ (*n* = 10) and to isolate them ex vivo (*n* = 3). The paracrine effects of bAVM-MSCs on endothelial cells (ECs) were assessed in an ex vivo model using MSC-derived supernatants (SNs) and the EC line HUVEC. Selected functional assays were validated using a second EC line (HCAEC).

**Results:**

In situ, cells with a MSC-like phenotype (CD90^pos^CD105^pos^CD73^pos^) were found in 7 out of 10 bAVM tissues analysed. Ex vivo, MSCs were isolated from fresh bAVM samples and were subsequently characterized according to the ISCT^®^ criteria. The bAVM-MSC SNs had no effect on the ECs’ migration, but promoted the proliferation of the ECs. The strongest stimulatory effect of the bAVM-MSC SNs was observed regarding the ECs’ tubulogenesis. Additionally, the bAVM-MSC SN induced a partial endothelial-to-mesenchymal transition in ECs.

**Conclusions:**

These findings indicate that bAVMs contain tissue-resident MSCs, which can potentially modulate the biology and functions of the ECs in the bAVM microenvironment. Thus, MSCs may play critical roles in the pathophysiology and the progression of this disease.

**Graphical Abstract:**

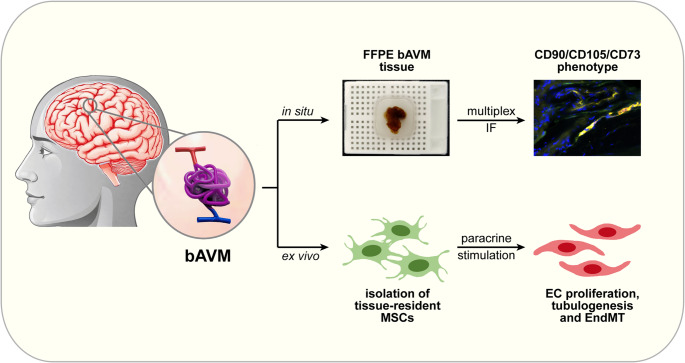

**Supplementary Information:**

The online version contains supplementary material available at 10.1007/s12015-025-10937-1.

## Introduction

Brain arteriovenous malformations (bAVMs) are vascular abnormalities characterized by tangles of dysplastic cerebral arteries and veins, without an intervening capillary bed. The overall incidence of bAVMs across population-based studies is relatively low, ranging from 1.10 to 1.42 cases per 100,000 indviduals [1]. However, due to their risk of rupture and subsequent intracranial hemorrhage, the affected patients can reach 1-year case fatality rates of 12% and poor outcome rates of up to 40% [2]. Although initially considered to be strictly congenital, more recent evidence indicates that bAVMs are dynamic, biologically active angiogenic and inflammatory lesions [3]. Consequently, understanding the cellular and molecular interactions that occur in the bAVM microenvironment may open new avenues for the management of this disease.

Mesenchymal stem/stromal cells (MSCs) were originally identified in the bone marrow, but are meanwhile known to reside in a variety of organs and tissues [4]. These cells are phenotypically characterized by the expression of CD90, CD105 and CD73 on the cell surface, with the concomitant absence of hematopoetic cell markers [5]. The “stemness” of MSCs results from their ability to undergo lineage-specific differentiation including, but not limited to, adipogenic, chondrogenic and osteogenic lineages. Additionally, MSCs have potent immunomodulatory properties, but a low immunogenicity, due to the absence of surface MHC-I expression and almost negligible MHC-II expression [6]. It is therefore not surprising that the largest body of research involving MSCs focused on their application as cell-based therapeutics for various medical conditions, such as wound healing disorders [7], autoimmune diseases [8] and cancer [6]. This is also the case in the field of cerebrovascular diseases research. Specifically, multiple randomized and non-randomized clinical trials investigated the effect of transplanted MSCs on ischemic stroke-associated neurological deficits (for a recent meta-analysis see [9]). Furthermore, studies on animal models of cerebral aneurysms tested whether the intravenous administration of MSCs or of MSC-derived microvesicles could prevent the aneurysms’ formation [10] and rupture [11, 12]. Using the swine rete mirabile as an AVM model, Touma and colleagues additionally tested if hydrogel-embedded bone marrow-MSCs could induce microvascular occlusion of the nidus via endoluminal tissue growth [13].

The role of tissue-resident MSCs in the pathophysiology of bAVM and of other cerebrovascular diseases is, however, currently unknown. In this study, we aimed to: (1) isolate and characterize MSCs from human bAVM tissues and (2) determine their effects on critical steps of the angiogenic cascade, such as migration, proliferation and tubulogenesis of the endothelial cells. Additionally, we sought to explore the potential link between MSCs and the induction of endothelial-to-mesenchymal transition in bAVM.

## Materials and Methods

### Material

Details on all materials used in this study, including manufacturer and catalog number, are listed in the supplementary Table S1.

### Study Subjects

This study included only adult patients with neuroradiologically and histopathologically confirmed bAVMs. The in situ immunofluorescence analyses were performed on formalin-fixed paraffin-embedded (FFPE) tissues from 10 bAVM patients that had been stored at the Department of Neuropathology, University Hospital Magdeburg following the routine diagnostic procedure. Ethical approval for this set of studies was granted by the ethics committee of the Medical Faculty, Otto-von-Guericke University, which issued a waiver for informed written consent (RENOVA study, No. 94/20). The bAVM-derived MSCs were isolated from tissues of three bAVM patients, freshly collected during surgery. This set of studies was also approved by the local ethics committee, and informed written consent was obtained from each patient (NEUROVA study, No. 68/20).

### Isolation and Characterization of Tissue-Resident MSCs

Fresh bAVM tissues were mechanically and enzymatically dissociated using the Tumor Dissociation Kit for 1 h at 37 °C, according to the manufacturer’s protocol. The samples were re-suspended in cell culture medium (Dulbecco´s modified Eagle´s medium (DMEM) supplemented with 10% FBS Supreme and 1% penicillin– streptomycin) and subjected to a plastic adherence step overnight at 37 °C. The adhered cells were subsequently cultured at 37 °C until full confluence. In order to confirm the purity of the isolated MSCs and characterize the expression of MSC surface markers, the samples were stained with CD45-PE, CD73-APC, CD90-FITC, and CD105-PE-Vio 770 antibodies, as recommended by the manufacturer. The analysis was performed by flow cytometry with a BD FACSCanto II cytometer. To assess the tri-lineage differentiation potential, MSCs were stimulated with Mesenchymal Stem Cell Adipogenic, Chondrogenic and Osteogenic Differentiation media, according to the manufacturer’s protocol. Following differentiation, the samples were fixed with Saccomanno solution for 30 min at room temperature and then washed once with distilled water. For the adipogenic differentiation, the samples were incubated in 60% Isopropanol for 5 min at room temperature and then in a Sudan III alcoholic solution for another 5 min. For the chondrogenic differentiation, the spheroids were incubated in a 0.1% Alcian Blue solution pH 2.5 for 30 min at room temperature, followed by 3 short de-staining steps with 60% ethanol and 40% acetic acid. For the osteogenic differentiation, the samples were incubated in an Alizarin Red S pH 4.0 solution for 15 min at room temperature. All samples were subsequently washed with distilled water, and analyzed immediately by brightfield microscopy.

### Cell Lines and Conditioned Supernatants (SNs)

In this study, we used the endothelial cell lines HUVEC and HCAEC, which were a kind gift from the Department of Cardiology and Angiology, Otto-von-Guericke University Magdeburg, but are also commercially available. The conditioned supernatants (SNs) were prepared from MSCs incubated at a density of 5 × 10^5^ cells/mL in cell culture medium at 37 °C for 24 h. The SNs were cleared by centrifugation at 1,200 × g, aliquoted, and stored at − 20 °C until use.

### Immunofluorescence

Multiplex immunofluorescence was performed on FFPE tissues, which were initially cut into 4 μm sections, deparaffinized, and subjected to heat-induced epitope retrieval (HIER) in citrate buffer pH 6.0. The stainings were performed with the Opal-6-Plex Kit according to the manufacturer’s protocol and as previously shown [14]. The following primary antibodies were used: 0.048 µg/mL CD90 rabbit monoclonal, 0.77 µg/mL CD105 mouse monoclonal, and 0.3 µg/mL CD73 rabbit monoclonal antibodies. To determine the Ki67 proliferation index, HUVEC cells were seeded on glass coverslips and stimulated with conditioned SNs or medium control for 72 h at 37 °C. The cells were fixed and permeabilized using the BD Cytofix/Cytoperm™ Kit for 20 min at 4 °C and subsequently stained with 0.36 µg/mL mouse monoclonal Ki67 antibodies overnight at 4 °C. Secondary reactions were carried out for 1 h at room temperature using AlexaFluor^®^488-coupled antibodies. The coverslips were finally embedded in Vectashield mounting medium containing DAPI. All samples were analyzed using a BZ-X810 digital fluorescence microscope.

### Western Blot

MSCs and HUVEC cells were lysed on ice in diluted Cell Lysis Buffer containing protease/phosphatase inhibitors. Cell debris was removed by high-speed centrifugation, and the lysates were subsequently incubated with an SDS-loading buffer containing 4% glycerin, 0.8% SDS, 1.6% beta-mercaptoethanol and 0.04% bromophenol blue. The proteins were separated by SDS-PAGE followed by transfer to Roti^®^-Fluoro PVDF membranes. The membranes were blocked in a solution containing 5% milk powder and were then incubated with the following primary antibodies: 0.1 µg/mL rat monoclonal GAPDH, 0.11 µg/mL mouse monoclonal Vimentin, 0.007 µg/mL rabbit monoclonal α-SMA, and 0.1 µg/mL rat monoclonal betaActin overnight at 4 °C. Secondary reactions were performed for 1 h at room temperature using AlexaFluor^®^488-, AlexaFluor^®^555-, and AlexaFluor^®^647-coupled antibodies. All antibodies were diluted using the Signal Boost™ Immunoreaction Enhancer Kit. Signal detection was performed on a ChemoStar imaging system.

### Cytokine Analysis

The levels of IL-4, IL-6, TGFβ, TNFα and VEGF in the bAVM-MSC SNs were assessed using DuoSet^®^ ELISA kits according to the manufacturer’s protocol. The OD values (450/540 nm) were measured on a TECAN plate reader.

### Gelatin Zymography

To assess the levels of gelatinases (MMP2 and MMP9) in the bAVM-MSCs SNs, the supernatants were mixed with Zymogram Sample Buffer at a final concentration of 80 mM Tris pH 6.8, 1% SDS, 4% glycerol and 0.006% bromophenol blue. Proteins were separated by SDS-PAGE containing 0.2% gelatin 180 Bloom and then renatured in 2.5% Triton-X-100 for 1 h at room temperature. The enzymatic reaction was performed overnight at 37 °C in a buffer containing 50 mM Tris pH 7.5, 200 mM NaCl, 5 mM CaCl_2_ and 1% Triton-X-100. The gels were stained with a solution containing 0.5% Brilliant Blue G250, 30% methanol and 10% acetic acid for 1 h at room temperature. Finally, the gels were de-stained with 30% methanol and 10% acetic acid until the digested bands became visible.

### Proliferation Assay

The effect of bAVM-MSC SNs on the proliferation of HUVEC cells was assessed using a colorimetric BrdU assay kit according to the manufacturer’s instructions. Briefly, the HUVEC cells were seeded in 96-well plates at a density of 5 × 10^3^ cells/well, and were incubated with bAVM-MSC SNs or medium control for 72 h at 37 °C. The samples were subsequently incubated with BrdU overnight at 37 °C, followed by a fixation and DNA denaturation step for 30 min at room temperature. The BrdU was detected by monoclonal primary antibodies and peroxidase-conjugated secondary antibodies for 1 h and 30 min at room temperature, respectively. After addition of the TMB substrate and stopping of the colorimetric reaction, the OD values (450/550 nm) were measured on a TECAN plate reader.

### Migration Assay

The effect of bAVM-MSC SNs on the migration of HUVEC cells was assessed using the Oris™ system. Briefly, 96-well plates were coated with a matrix containing 1 mg/ml collagen I for 30 min at 37 °C. The cells were added to the wells and were allowed to adhere overnight at 37 °C in the presence of a stopper, which formed a “gap” in the middle of the well. Following the removal of the stopper, the cells were allowed to migrate in the cell-free area for 24 h. The degree of “gap-closure” (cell free area at 0 h vs. 24 h) was quantified with the ImageJ 1.48v software.

### Tube Formation Assay

The effect of bAVM-MSC SNs on the tubulogenesis of HUVEC and HCAEC was assessed using tube formation assays. To this end, the wells of a 96-well plate were coated with 50 µl undiluted Cultrex Basement Membrane Extract for 30 min at 37 °C, and the cells were seeded at a density of 1.5 × 10^4^/well. Following a 24 h incubation period at 37 °C, the number and length of segments and branches were quantified using the angiogenesis macro of the ImageJ 1.48v software.

### Analysis of Endothelial-to-Mesenchymal Transition (EndMT)

The effect of bAVM-MSC SNs on the EndMT of HUVEC cells was assessed by flow cytometric analysis of surface markers using VE-Cadherin-VioBlue and CD31-APC-Vio770 antibodies according to the manufacturer’s recommendation. Furthermore, the cells were fixed and permeabilized with the BD Cytofix/Cytoperm™ Kit for 20 min at 4 °C, and then stained for the intracellular expression of SNAIL (SNAI1) using CoraLite^®^Plus488-conjugated antibodies for 1 h at room temperature. Additionally, the expression levels of Vimentin were assessed by western blot as described above.

### Statistical Analysis

The data from all studies were statistically analyzed using the paired Student’s *t*-test. The level of significance was set at *p* ≤ 0.05 in all studies.

## Results

### bAVM Contain Tissue-Resident MSCs

**Fig. 1:**
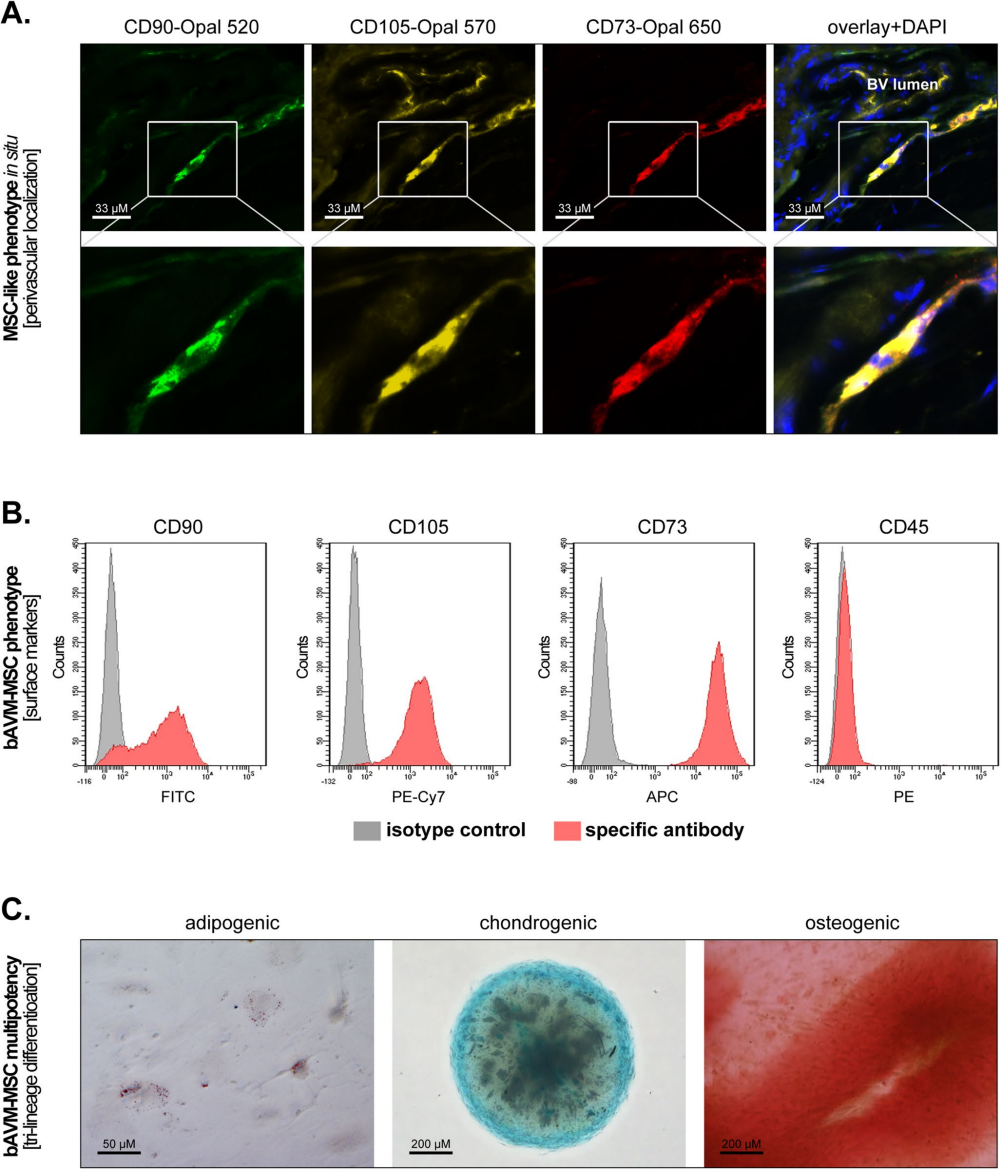
Characterization of bAVM-MSCs in situ and ex vivo. (**A**) Representative multiplex immunofluorescence micrographs of bAVM tissues stained against CD90 (green), CD105 (yellow) and CD73 (red) showing cells positive for all three markers in the perivascular region. The nuclei were counterstained with DAPI (blue). BV = blood vessel. (**B**) Representative flow cytometric analysis of the surface markers CD90, CD105, CD73 and CD45. The specific stainings are indicated by red histograms and the respective isotype controls by grey histograms. (**C**) Representative micrographs of the tri-lineage differentiation studies showing Sudan III-positive lipid vesicles (left panel), Alcian blue-positive cartilage (middle panel) and Alizarin red-positive mineralized bone matrix (right panel)

To determine whether bAVM may contain tissue-resident MSCs, we stained FFPE tissues from 10 bAVM patients by multiplex immunofluorescence against the MSC markers CD90, CD105 and CD73. In the majority of samples (7 out of 10), we could identify cells that co-expressed all three markers. These cells mostly formed “chain”-like structures in the proximity of blood vessels (Fig. 1A), but some isolated cells appeared to be localized directly in the vessels’ Tunica externa (suppl. Figure S1A).

Next, we aimed to isolate and characterize these cells ex vivo. To this end, we used fresh tissues from three different bAVM patients. The phenotypic characterization of the isolated cells by flow cytometry demonstrated an uniform positivity for CD105 and CD73. CD90 was expressed by the majority of the cells, while CD45 was– as expected– negative (Fig. 1B). Furthermore, fluorescent western blot analyses demonstrated that the isolated cells were positive for the mesenchymal markers Vimentin and alpha-SMA (suppl. Figure S1B). Additionally, we performed tri-lineage differentiation studies using specialized stimulation media. The results showed that the cells succesfully differentiated into the adipogenic, chondrogenic and osteogenic lineages upon stimulation, though it should be mentioned that the adipogenic differentiation was relatively weak compared to the other two lineages (Fig. 1C). Taken together these findings indicate that bAVM contain tissue-resident MSCs, which can be isolated and cultured ex vivo.

### bAVM-MSCs Enhance Endothelial Proliferation and Tubulogenesis

**Fig. 2:**
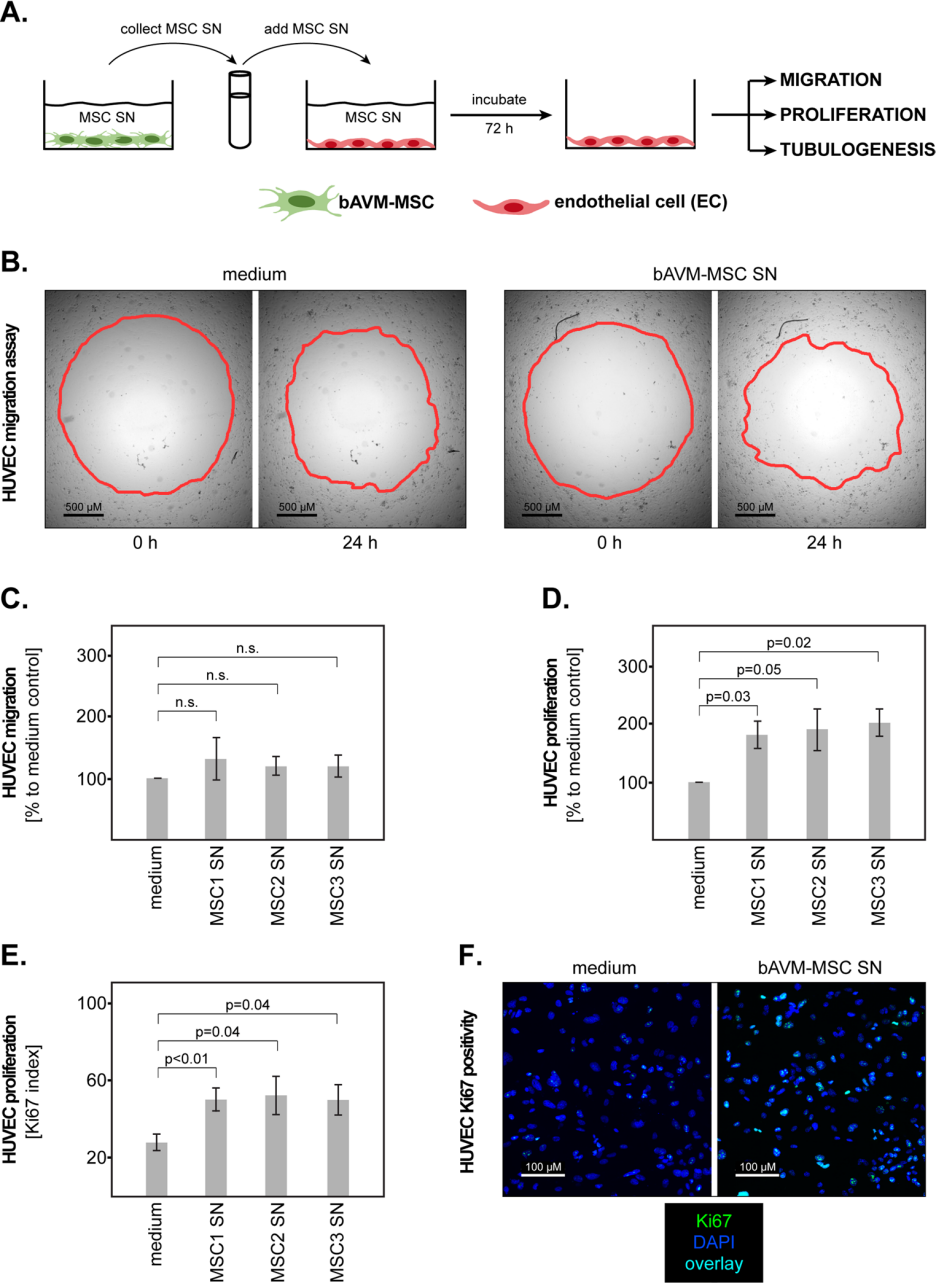
Effect of bAVM-MSCs on the EC migration and proliferation. (**A**) Experimental design of the EC stimulation and analysis. **(B)** Representative micrographs of HUVEC migration at 0 h (pre-migration status) and 24 h (post-migration status). The red lines mark the closure of the “gap“, indicating the degree of cell migration. (**C**) The bAVM-MSC SN do not significantly enhance the migration of HUVEC cells. Shown are the means and S.D. of three independent experiments. Statistical analysis was performed with the paired t-test. (**D**) bAVM-MSC SN significantly increase the proliferation of the HUVEC cells, as measured by the BrdU assay. Shown are the means and S.D. of three independent experiments. Statistical analysis was performed with the paired t-test. (**E**) bAVM-MSC SNs significantly increase the Ki67 proliferation index in HUVEC cells. Shown are the means and S.D. of three independent experiments. Statistical analysis was performed with the paired t-test. (**F**) Representative micrographs showing the expression of nuclear Ki67 (green) in HUVEC cells stimulated with bAVM-MSC SN (right panels) versus medium control (left panels). The cell nuclei were counterstained with DAPI (blue)

Next, we sought to determine whether bAVM-MSCs release soluble factors that may affect the angiogenic processes in endothelial cells. To this end, we produced conditioned supernatants (SN) from our isolated MSCs and used them to stimulate endothelial cells (EC) for 72 h. We subsequently investigated several critical steps of the angiogenic cascade, namely migration, proliferation and tubulogenesis of the ECs (Fig. 2A). Incubation of the ECs in regular cell culture medium instead of MSC SN served as control in all assays. For this set of studies we used the HUVEC cell line as an EC model.

The migration of the ECs was assessed at 24 h post-stimulation using the Oris™ assay (Fig. 2B). Subsequent analysis of the “gap“-closure with the ImageJ software revealed no significant differences between HUVEC stimulated with bAVM-MSC SNs and those from the medium control group (Fig. 2C). To assess EC proliferation, we perfomed BrdU assays. The results showed that all three bAVM-MSC SNs significantly increased the proliferation of the HUVEC cells (Fig. 2D). Additionally, we found that all samples stimulated with bAVM-MSC SNs had a significantly increased nuclear positivity for the proliferation marker Ki67 (Ki67 index) compared to the medium control group (Fig. 2E-F).

**Fig. 3:**
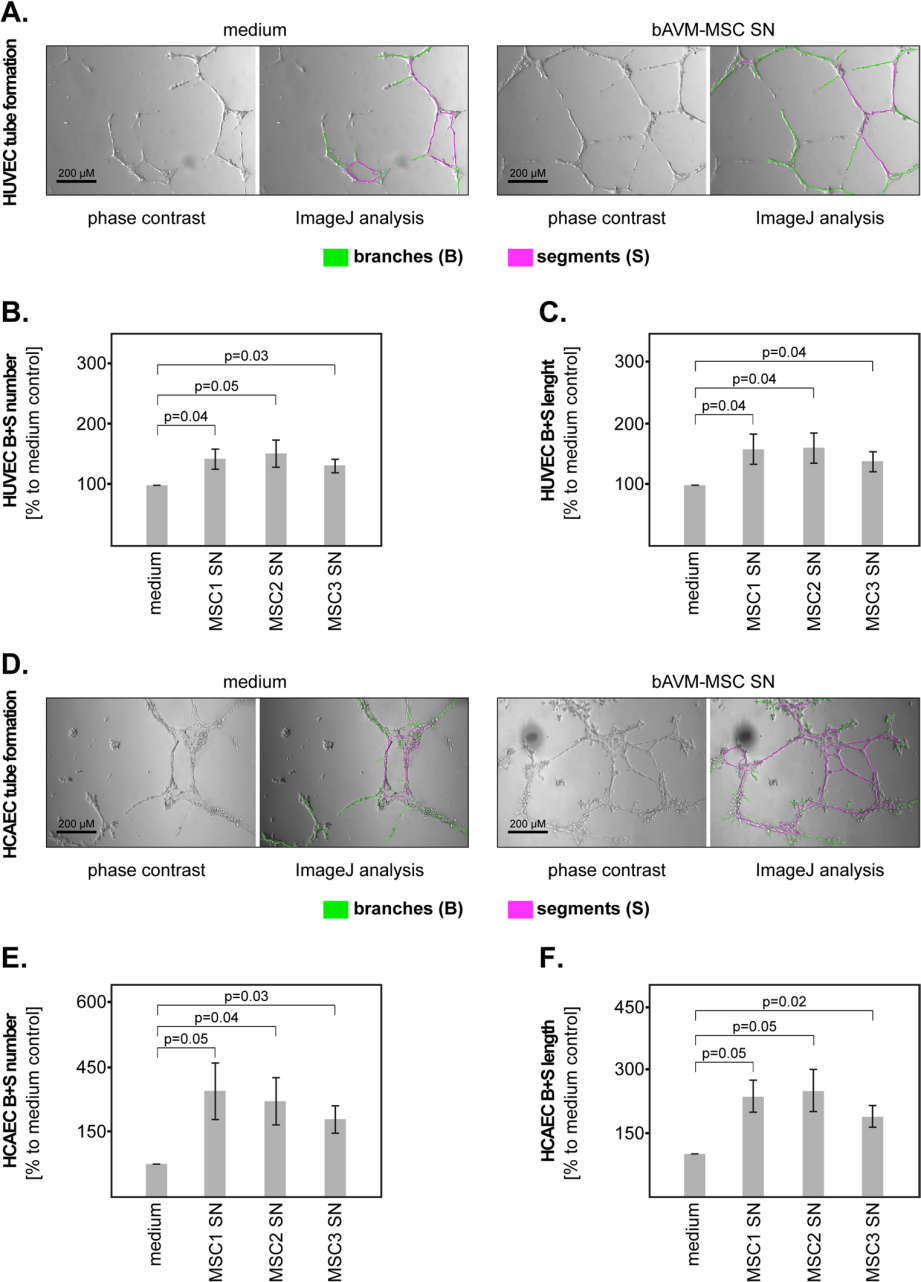
Effect of bAVM-MSCs on the EC tubulogenesis. (**A**) ImageJ analysis of the tube formation assays in HUVEC cells stimulated with bAVM-MSC SN (right panels) versus medium control (left panels). The branches are indicated in green and the segments in magenta. All three bAVM-MSC SN significantly increased the (**B**) number and (**C**) lenght of branches and segments in HUVEC cells compared to medium control. (**D**) ImageJ analysis of the tube formation assays in HCAEC cells stimulated with bAVM-MSC SN (right panels) versus medium control (left panels). The branches are indicated in green and the segments in magenta. All three bAVM-MSC SN significantly increased the (**E**) number and **(F**) lenght of branches and segments in HCAEC cells compared to medium control. Shown are the means and S.D. of three independent experiments. Statistical analysis was performed with the paired t-test

To assess EC tubulogenesis, we performed tube formation assays at 24 h post-stimulation, and analysed the development of branches and segments using the ImageJ software (Fig. 3A). The results showed that all three bAVM-MSC SNs induced a significant increase of the number of branches and segments in HUVEC cells compared to medium control (Fig. 3B). Furthermore, the length of the branches and segments was significantly higher in HUVEC stimulated with bAVM-MSC SNs (Fig. 3C). Since the bAVM-MSC SNs had a robust effect on the tube formation of HUVEC cells, we sought to validate these findings using a second EC line (HCAEC). The stimulation of HCAEC cells with bAVM-MSC SN and the tube formation assays were performed in a similar manner as for HUVEC (Fig. 3D). The results showed that both the number (Fig. 3E) and the length (Fig. 3F) of branches and segments were significantly increased in HCAEC stimulated with bAVM-MSC SN compared to medium control.

Taken together, these findings indicate that bAVM-MSCs release soluble factors, which promote the angiogenesis of ECs. Consequently, we tested whether the bAVM-MSC SNs contained pro-angiogenic factors and found significant amounts of VEGF (suppl. Figure S2A) and of gelatinases, in particular MMP9 (suppl. Figure S2B). Other factors with potential pro-angiogenic functions were present only in modest amounts (IL-6) or were under the detection limit of the respective assays (TGFβ, TNFα, IL-4) (suppl. Figure S2A).

### bAVM-MSCs Induce a Partial EndMT in Endothelial Cells

**Fig. 4:**
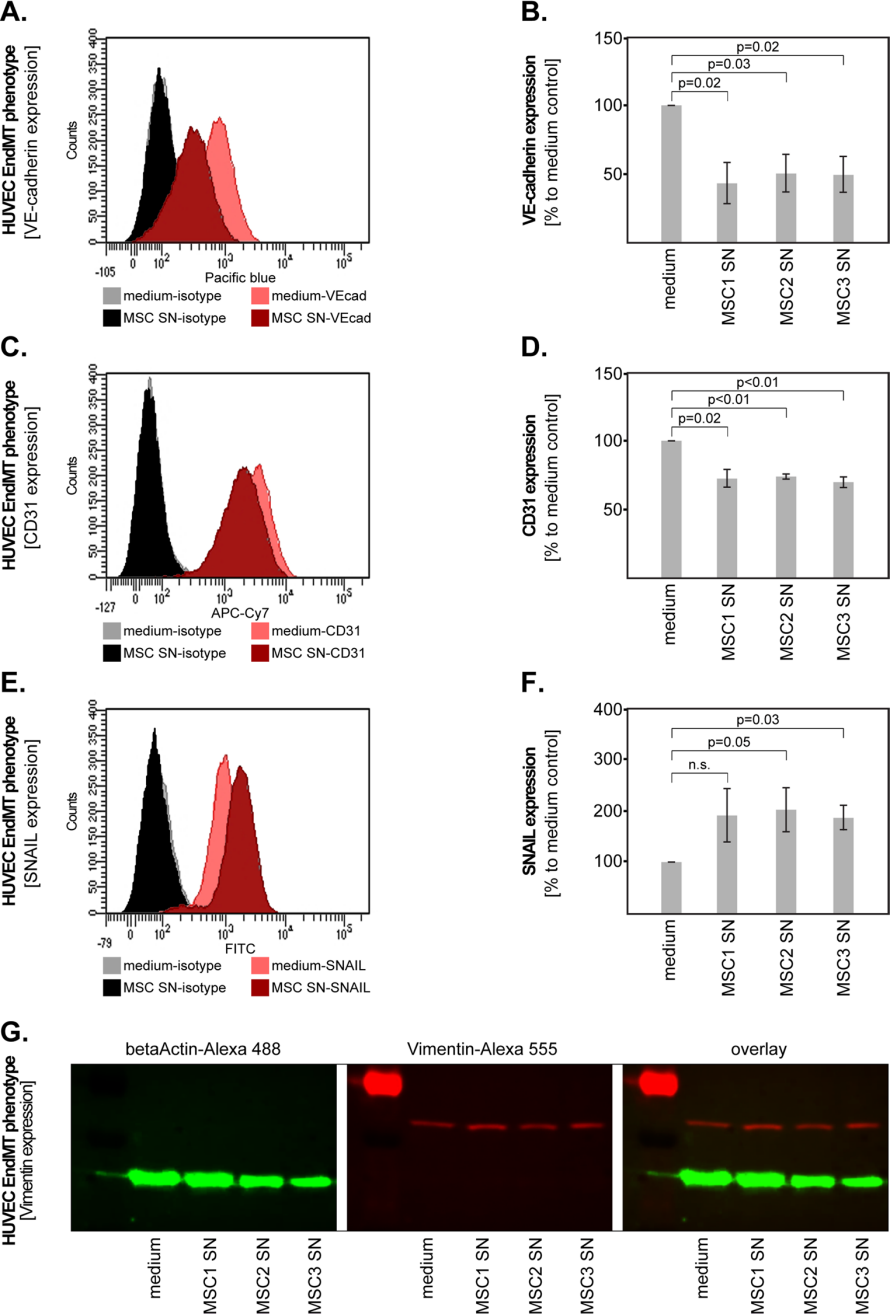
Effect of bAVM-MSCs on the EndMT phenotype of EC cells. Representative flow cytometric analysis of (**A**) VE-cadherin, (**C**) CD31 and (**E**) SNAIL in HUVEC cells stimulated with bAVM-MSC SN or medium control. The bAVM-MSC SNs significantly downregulated the expression of the endothelial surface markers (**B**) VE-cadherin and (**D**) CD31 and (**F**) upregulated the expression of the transcription factor SNAIL in HUVEC cells. Shown are the means and S.D. of three independent experiments. Statistical analysis was performed with the paired t-test. (**G**) Representative fluorescent western blot of HUVEC cells stimulated with bAVM-MSC SNs versus medium control showing the expression of Vimentin (red). Beta-Actin (green) was used as loading control

In the final set of studies, we aimed to determine whether bAVM-MSCs can potentially induce endothelial-to-mesenchymal transition (EndMT) in ECs. To this end, we stimulated the HUVEC cells as previously described in Fig. 2A and analysed the expression of endothelial surface markers (VE-cadherin, CD31), transcription factors (SNAIL), and mesenchymal markers (N-cadherin, Vimentin, alpha-SMA), respectively. The results showed that HUVEC stimulated with bAVM-MSC SNs had a significantly lower expression of VE-cadherin (Fig. 4A-B) and CD31 (Fig. 4C-D) compared to the medium control group. Furthermore, the expression of SNAIL was upregulated upon stimulation with all bAVM-MSC SNs, and statistical significance was reached with two out of three SNs (Fig. 4E-F). In contrast, the analysis of the mesenchymal markers was not very conclusive. Specifically, the (weak) baseline expression of Vimentin was not consistently increased in HUVEC stimulated with bAVM-MSC SNs (Fig. 4G), while N-cadherin and alpha-SMA could not be detected in any sample, neither by western blot nor by flow cytometry. Taken together these findings indicate that bAVM-MSC may induce a partial EndMT in ECs.

## Discussion

Tissue-resident MSCs have been identified in a variety of organs and medical conditions, but their role in the pathophysiology of bAVM is currently unknown. To the best of our knowledge, this study is the first to isolate and characterize MSCs from human bAVM tissues. Since the in situ identification of MSCs is particularly challenging (due to the lack of unique markers), their ex vivo characterization based on phenotype and multipotency remains the “gold standard” in the MSC research field. In line with the ISCT^®^ criteria for defining MSCs [5], our bAVM-derived cells were adherent on plastic under standard culture conditions, had a CD90^pos^CD105^pos^CD73^pos^CD45^neg^ phenotype, and underwent tri-lineage differentiation (adipogenic, chondrogenic and osteogenic) ex vivo. Interestingly, using sequential multiplex immunofluorescence, we also found cells with a CD90^pos^CD105^pos^CD73^pos^ phenotype in the majority of the bAVM tissues analysed. It should be mentioned at this point that most previous studies, which attempted to identify human MSCs in situ, did not assess the simultaneous expression of these three major MSC markers. Instead, the tissues were analysed either by single-color immunohistochemistry/immunofluorescence against CD90, CD73, CD44 or CD146, respectively [15, 16], or by double immunofluorescence against CD90/CD105 [17]. Therefore, it is presently unclear if MSC express the same surface phenotype in situ as they do under cell culture conditions. However, in an elegant study using chipcytometry-based multiplex immunofluorescence, Consentius and colleagues identified cells with a CD90^pos^CD105^pos^CD73^pos^ phenotype in human placenta [18]. Together with our own data, these findings indicate that multiplex methods using this marker combination are suitable to identify tissue-resident MSCs (or at least a subpopulation of them) also in situ.

While the exact phenotype of tissue-resident MSCs may require further characterization, there seems to be a general consensus that MSCs are localized in the close proximity of blood vessels, or even directly in the vessels’ wall (reviewed in [19–22]). This phenomenon was observed in our study as well. Taking into consideration this particular histological localization, it is likely that MSC-derived factors would easily reach the endothelial cells (ECs) thereby affecting their biology and, ultimately, angiogenesis. Since the angiogenic cascade is very complex, our study focused only on selected steps in this process, specifically on the migration, proliferation and tubulogenesis of the ECs (reviewed in [23]). Our data showed that bAVM-MSCs had no effect on EC migration, but enhanced EC proliferation, as indicated by the BrdU assay and the analysis of the Ki67 index. Importantly, we observed a robust effect of the bAVM-MSCs SNs on EC tubulogenesis, as indicated by the significantly increased number and length of branches/segments in two different cell line models. These findings suggest that tissue-resident MSCs have pro-angiogenic functions in the microenvironment of bAVM.

MSC-induced angiogenesis has been mainly studied in the context of therapeutic approaches for regenerative medicine using bone marrow-derived MSCs (recently reviewed in [24]). While much less data is currently available on tissue-resident MSCs, similar mechanisms may apply here as well [25]. One potential mechanism of MSC-induced angiogenesis is by means of their ability to differentiate into ECs [26–29]. MSCs may additionally interact with the ECs via direct cell-cell contact, but it remains controversial whether this interaction promotes or inhibits angiogenesis [30–32]. However, considering their relative scarcity and histological localization, tissue-resident MSCs are more likely to affect other cells in the microenvironment through secreted factors rather than through differentiation or direct physical contact. This mode of action was also mimicked by our study’s experimental design. Although MSCs can release a plethora of factors (reviewed in [33]), multiple studies demonstrated a critical role of VEGF in MSC-induced angiogenesis [34–36]. The analysis of our bAVM-MSC SNs by ELISA found VEGF levels between 480 and 839 pg/ml, which were comparable to the ones shown to enhance HUVEC tubulogenesis in a previous study [36]. Since VEGF is also a major factor in the bAVM pathophysiology [37–41], it is feasible that MSCs may contribute to the progression of bAVMs via this mechanism. Additionally, the bAVM-MSCs released significant levels of MMP9, which is known to mobilize VEGF from the extracellular matrix, thereby potentially enhancing its bioavailability in the bAVM microenvironment.

Finally, our findings suggest a link between bAVM-MSCs and the induction of endothelial-to-mesenchymal transition (EndMT) in ECs. Although the ECs stimulated with MSC-derived factors did not undergo a complete EndMT in our study, they did exhibit critical features associated with this process, such as destabilization of the adherens junctions (as indicated by the downregulation of VE-cadherin and CD31/PECAM1) as well as the upregulation of the transcription factor SNAIL (reviewed in [42, 43]). The role of EndMT in the bAVM pathophysiology has recently started to emerge [44], but it is still poorly understood thus far. In 2019, Yao and colleagues reported for the first time that human bAVM tissues expressed EndMT markers, both at RNA and protein level [45]. This abberant expression of endothelial and mesenchymal markers in bAVMs was confirmed shortly thereafter by two other independent groups [46, 47]. To date, only one group addressed the cellular and molecular mechanisms governing the EndMT process in bAVM, and identified a mechanism involving the release of exosomes by rare bAVM-ECs expressing the KRAS^G12D^ mutation. These exosomes contained high levels of miR-3131, which induced EndMT in HUVEC cells potentially via the ERK-TGFβ-BMP-SMAD4 pathway [48, 49]. Our findings indicate that, apart from the KRAS^G12D^-ECs, MSCs may also contribute to the EndMT process in the microenvironment of bAVMs. The exact MSC-derived factors responsible for this phenomenon remain, however, to be characterized in future studies.

In conclusion, our study provides first evidence that human bAVMs contain tissue-resident MSCs. These cells modulate the biology and functions of the ECs in a paracrine manner, by inducing their proliferation, tubulogenesis and the EndMT switch. Therefore, MSCs may represent novel players in the bAVM microenvironment, with potentially critical roles in the pathophysiology and progression of this disease.

## Supplementary Information

Below is the link to the electronic supplementary material.

Supplementary file1 (PDF 487 KB)

## Data Availability

All raw data and material used in this study can be obtained from the corresponding author upon reasonable request.
