# Supplementary material for: Tissue-Resident Mesenchymal Stem/stromal Cells (MSC) Modulate the Angiogenic Processes in Brain Arteriovenous Malformations (bAVM)
Source: Stem Cell Rev Rep. 2025 Jul 17;21(7):2327–38. doi: 10.1007/s12015-025-10937-1 (PMC12408775; doi:10.1007/s12015-025-10937-1)
Supplement: Supplementary file 1 — Supplementary file1 (PDF 487 KB) [file 12015_2025_10937_MOESM1_ESM.pdf]

suppl. Table S1

	Product Name	Manufacturer	Cat. No.
Antibodies	AlexaFluor®488 anti-mouse IgG F(ab)2	Cell Signaling Technology, Frankfurt am Main, Germany	4408
	AlexaFluor®488 anti-rat IgG F(ab)2	Cell Signaling Technology, Frankfurt am Main, Germany	4416
	AlexaFluor®555 anti-mouse IgG F(ab)2	Cell Signaling Technology, Frankfurt am Main, Germany	4409
	AlexaFluor®647 anti-rabbit IgG F(ab)2	Cell Signaling Technology, Frankfurt am Main, Germany	4414
	BetaActin rat monoclonal	Biolegend, San Diego, CA, USA	664801
	CD31-APC-Vio770 anti-human REAfinity	Miltenyi Biotech, Bergisch Gladbach, Germany	130-117-318
	CD45-PE, anti-human REAfinity	Miltenyi Biotech, Bergisch Gladbach, Germany	130-110-632
	CD73-APC (REA804) anti-human REAfinity	Miltenyi Biotech, Bergisch Gladbach, Germany	130-112-061
	CD73 (D7F9A) rabbit monoclonal	Cell Signaling Technology, Frankfurt am Main, Germany	13160
	CD90-FITC anti-human REAfinity	Miltenyi Biotech, Bergisch Gladbach, Germany	130-114-901
	CD90 (Thy1) rabbit monoclonal	Cell Signaling Technology, Frankfurt am Main, Germany	13801
	CD105/Endoglin (3A9) mouse monoclonal	Cell Signaling Technology, Frankfurt am Main, Germany	14606
	CD105-PE-Vio770 anti-human REAfinity	Miltenyi Biotech, Bergisch Gladbach, Germany	130-112-325
	CoraLite®Plus 488 rabbit IgG1 isotype control recombinant antibody	Proteintech, Rosemont, IL, USA	CL488-98136
	GAPDH Ab (W17079A) rat monoclonal	Biolegend, San Diego, CA, USA	607901
	REA control antibody, human, APC	Miltenyi Biotech, Bergisch Gladbach, Germany	130-113-446
	REA control antibody, human, APC-Vio770	Miltenyi Biotech, Bergisch Gladbach, Germany	130-113-447
	REA control antibody, human, FITC	Miltenyi Biotech, Bergisch Gladbach, Germany	130-113-449
	REA control antibody, human, PE	Miltenyi Biotech, Bergisch Gladbach, Germany	130-113-450
	REA control antibody, human, PE-Vio770	Miltenyi Biotech, Bergisch Gladbach, Germany	130-113-452
	REA control antibody, human, VioBlue	Miltenyi Biotech, Bergisch Gladbach, Germany	130-113-454
	Ki67 (8D5) mouse monoclonal	Cell Signaling Technology, Frankfurt am Main, Germany	9449

	SNAI1-CoraLite®Plus 488 recombinant antibody	Proteintech, Rosemont, IL, USA	CL488-81584-4
	VE-cadherin (CD144)-VioBlue human	Miltenyi Biotech, Bergisch Gladbach, Germany	130-100-725
	Vimentin mouse monoclonal	Proteintech, Rosemont, IL, USA	60330-1-Ig
	α-SMA (Smooth Muscle Action) (D4K9N) rabbit monoclonal	Cell Signaling Technology, Frankfurt am Main, Germany	19245
**Kits**	BD Perm/Wash™ kit	BD Biosciences, Heidelberg, Germany	554714
	BrdU Cell Proliferation Assay Kit	Merck-Millipore, Burlington, MA, USA	2750
	Human IL-4 DuoSet ELISA	RnD Systems, Minneapolis, MN, USA	DY204-05
	Human IL-6 DuoSet ELISA	RnD Systems, Minneapolis, MN, USA	DY206-05
	Human TGF-beta 1 DuoSet ELISA	RnD Systems, Minneapolis, MN, USA	DY240-05
	Human TNF-alpha DuoSet ELISA	RnD Systems, Minneapolis, MN, USA	DY210-05
	Human VEGF DuoSet ELISA	RnD Systems, Minneapolis, MN, USA	DY293B-05
	Opal^{TM} 6-Plex Detection Kit	Akoya Biosciences, Marlborough, MA, USA	NEL811001KT
	Oris^{TM} 3D Embedded Invasion Assay Kit	Platypus Technologies, Madison, WI, USA	EIA1
	Signal Boost™ Immunoreaction Enhancer Kit	Merck-Millipore, Burlington, MA, USA	407207-1KIT
	Tumor Dissociation Kit, human	Miltenyi Biotech, Bergisch Gladbach, Germany	130-095-929
**Chemicals and disposable material**	Acetic acid	Carl Roth, Karlsruhe, Germany	3738.2
	Alcian blue 0,1%, pH 2,5 with acetic acid	Morphisto, Frankfurt, Germany	11490.00250
	Alizarin-red S, pH 4.0	Morphisto, Frankfurt, Germany	13158.00250
	Ammonium peroxydisulphate	Carl Roth, Karlsruhe, Germany	9592.3
	Aqua B. Braun	B. Braun SE, Melsungen, Germany	0082479E
	Beta-mercaptoethanol	Carl Roth, Karlsruhe, Germany	4227.3
	Brilliant blue G 250	Carl Roth, Karlsruhe, Germany	9598.1
	Bromophenol blue	Carl Roth, Karlsruhe, Germany	A512.2
	Citrate buffer pH 6	Sigma Aldrich, St. Louis, MO, USA	C9999-1000ML
	Cell lysis buffer (10x)	Cell Signaling Technology, Frankfurt am Main, Germany	9803
	Cultrex Reduced Growth Factor Basement Membrane Extract	Bio-Techne, Minneapolis, MN, USA	3433-005-01
	EDTA	Carl Roth, Karlsruhe, Germany	8040.3
	Ethanol	Carl Roth, Karlsruhe, Germany	K928.3
	FBS Supreme	Pan Biotech, Aidenbach, Germany	P30-3031
	Gelatine 180 bloom	Carl Roth, Karlsruhe, Germany	4274.4
	Glycerin	Carl Roth, Karlsruhe, Germany	3783.1

	Mesenchymal Stem Cell Adipogenic Differentiation Medium 2	PromoCell, Heidelberg, Germany	C-28016
	Mesenchymal Stem Cell Chondrogenic Differentiation Medium 2	PromoCell, Heidelberg, Germany	C-28012
	Mesenchymal Stem Cell Osteogenic Differentiation Medium 2	PromoCell, Heidelberg, Germany	C-28013
	Methanol	Carl Roth, Karlsruhe, Germany	0082.2
	Milk powder	Carl Roth, Karlsruhe, Germany	T145.2
	Oris Collagen I (rat Tail)	Platypus Technologies, Madison, WI, USA	24B2801
	Pageruler™ Plus prestained Protein Ladder	Thermo Fisher Scientific, Waltham, MA, USA	26619
	Penicillin-Streptomycin	Thermo Fisher Scientific, Waltham, MA, USA	15140122
	Protease/phosphatase inhibitor cocktail (100x)	Cell Signaling Technology, Frankfurt am Main, Germany	5872
	ROTI®Cell 10x PBS	Carl Roth, Karlsruhe, Germany	9150.1
	Roti®Cell DMEM high glucose	Carl Roth, Karlsruhe, Germany	9007.1
	Roti®Fluoro PVDF Transfer Membrane	Carl Roth, Karlsruhe, Germany	2803.1
	Rotiphorese®Gel 40 (29:1)	Carl Roth, Karlsruhe, Germany	A515.1
	Saccomanno fixation solution	Morphisto, Frankfurt, Germany	1388100250
	SDS ultra pure	Carl Roth, Karlsruhe, Germany	2326.4
	Sudan III, alcoholic	Morphisto, Frankfurt, Germany	10396.00250
	TBS (10x)	Thermo Fisher Scientific, Waltham, MA, USA	10776834
	TEMED	Carl Roth, Karlsruhe, Germany	2367.3
	Tris	Carl Roth, Karlsruhe, Germany	4855.2
	Triton X-100	Carl Roth, Karlsruhe, Germany	3051.4
	Tween 20®	Carl Roth, Karlsruhe, Germany	9127.1
	VECTASHIELD Vibrance® Mounting Medium with DAPI	Vector Laboratories, Burlingame, CA, USA	H-1800
Software	BD FACSDiva™	BD Biosciences, Heidelberg, Germany	n.a.
	ImageJ 1.48v	NIH, Bethesda, MD, USA	n.a.
	Magellan™ 7.2	Tecan Life Sciences, Männedorf, Switzerland	n.a.
Equipment	BD FACSCanto II flow cytometer	BD Biosciences, Heidelberg, Germany	n.a.
	BZ-X810 fluorescence microscope	Keyence, Neu-Isenburg, Germany	n.a.
	ChemoStar Imaging system	Intas Science Imaging, Göttingen, Germany	n.a.
	TECAN plate reader	Tecan Life Sciences, Männedorf, Switzerland	n.a.

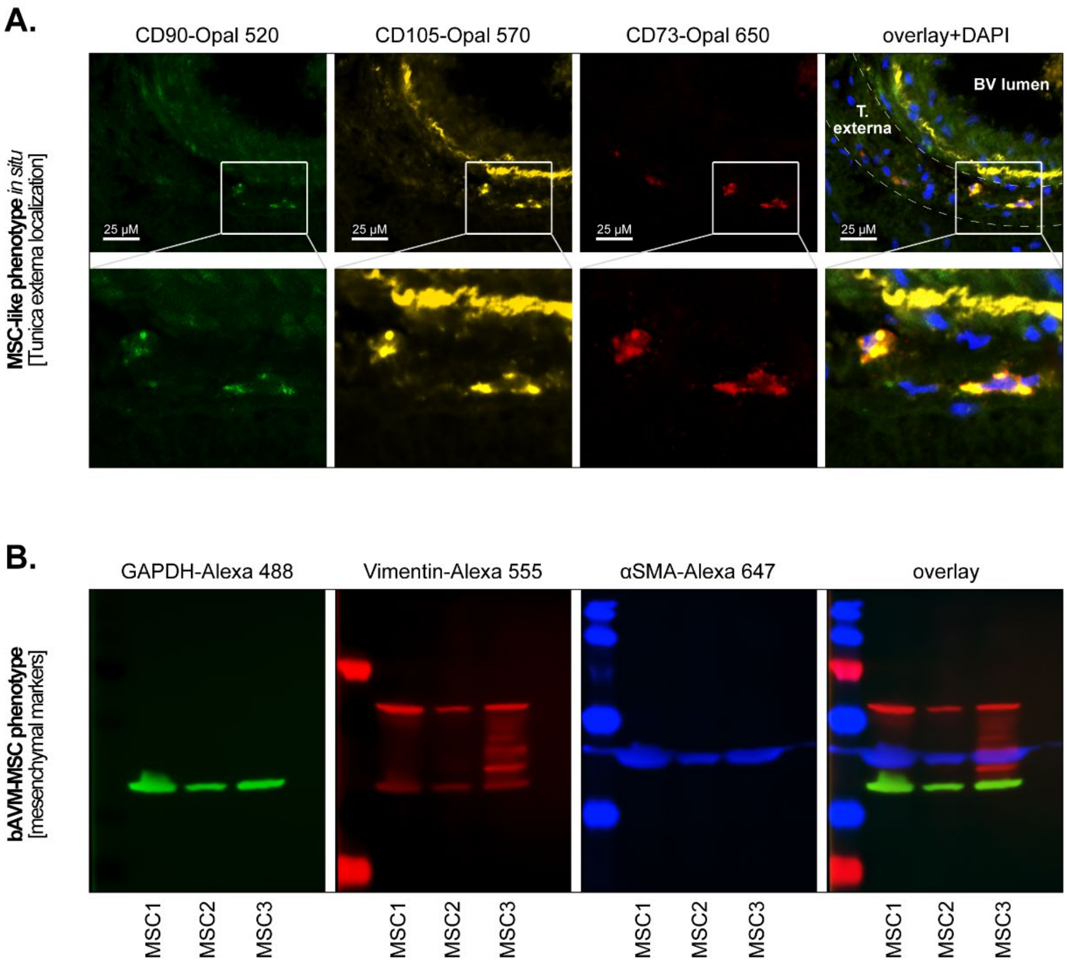

suppl. Figure S1. Characterization of bAVM-MSCs *in situ* and *ex vivo*. (A) Representative multiplex immunofluorescence micrographs of bAVM tissues stained against CD90 (green), CD105 (yellow) and CD73 (red) showing cells positive for all three markers in Tunica externa (T. externa) of the blood vessels (BV). **(B)** Fluorescent western blot analysis of all three bAVM-MSCs showing positivity for Vimentin (red) and alpha-SMA (blue). GAPDH (green) was used as loading control.

A.

ELISA SAMPLE	VEGF [pg/ml]	IL-6 [pg/ml]	TGFß [pg/ml]	TNFα [pg/ml]	IL-4 [pg/ml]
MSC1 SN	480	135	<31	<15	<31
MSC2 SN	839	134	<31	<15	<31
MSC3 SN	674	70	<31	<15	<31

B.

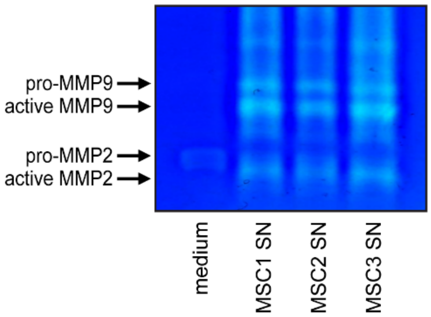

suppl. Figure S2. Pro-angiogenic factors in the bAVM-MSC SNs. (A) The levels of VEGF, IL-6, TGFβ, TNFα and IL-4 in each bAVM-MSC SN as determined by ELISA **(B)** Representative gelatin zymography showing the levels of gelatinases (MMP2 and MMP9) in all three bAVM-MSC SNs.
